# Diaphragm dysfunction and peripheral muscle wasting in septic shock patients: Exploring their relationship over time using ultrasound technology (the MUSiShock protocol)

**DOI:** 10.1371/journal.pone.0266174

**Published:** 2022-03-28

**Authors:** Ivo Neto Silva, José Alberto Duarte, Aurélie Perret, Nicolas Dousse, Hannah Wozniak, Bernardo Bollen Pinto, Raphaël Giraud, Karim Bendjelid

**Affiliations:** 1 Intensive Care Division, Department of Acute Medicine, Geneva University Hospitals, Geneva, Switzerland; 2 Faculty of Medicine, University of Geneva, Geneva, Switzerland; 3 Geneva Hemodynamic Research Group, Geneva, Switzerland; 4 Research Centre in Physical Activity (CIAFEL), Faculty of Sports, University of Porto, Porto, Portugal; 5 Anaesthesiology Division, Department of Acute Medicine, Geneva University Hospitals, Geneva, Switzerland; Yale University, UNITED STATES

## Abstract

**Background:**

Intensive Care Unit (ICU) patients are known to lose muscle mass and function during ICU stay. Ultrasonography (US) application for the assessment of the skeletal muscle is a promising tool and might help detecting muscle changes and thus several dysfunctions during early stages of ICU stay. *MUSiShock* is a research project aiming to investigate structure and function of diaphragm and peripheral muscles using ultrasound techniques in septic shock patients, and to assess their relevance in several clinical outcomes such as the weaning process.

**Methods and design:**

This is a research protocol from an observational prospective cohort study. We plan to assess eighty-four septic shock patients during their ICU stay at the following time-points: at 24 hours of ICU admission, then daily until day 5, then weekly, at extubation time and at ICU discharge. At each time-point, we will measure the *quadriceps rectus femoris* and *diaphragm* muscles, using innovative US muscle markers such as Shear-Wave Elastography (SWE). In parallel, the Medical Research Council (MRC) sum score for muscle testing and the Airway occlusion pressure (P_0.1_) will also be collected. We will describe the association between SWE assessment and other US markers for each muscle. The association between the changes in both *diaphragm* and *rectus femoris* US markers over time will be explored as well; finally, the analysis of a combined model of one diaphragm US marker and one limb muscle US marker to predict weaning success/failure will be tested.

**Discussion:**

By using muscle ultrasound at both diaphragm and limb levels, *MUSiShock* aims to improve knowledge in the early detection of muscle dysfunction and weakness, and their relationship with muscle strength and MV weaning, in critically ill patients. A better anticipation of these short-term muscle structure and function outcomes may allow clinicians to rapidly implement measures to counteract it.

**Trial registration:**

ClinicalTrials.gov, NCT04550143. Registered on 16 September 2020.

## Introduction

The diaphragm is the most important respiratory muscle as it plays a key role in lung ventilation. Under mechanical ventilation (MV), the diaphragm function is totally or partially replaced by artificial respiratory support. Weaning from MV “covers the entire process of liberating the patient from mechanical support and from the endotracheal tube” [1]. This process can be negatively affected by a diaphragm dysfunction leading to a higher probability of weaning failure [2], and thus worst clinical results. It can be observed at both early [3] and later stages of the critical illness experience [4], as a consequence of the combination of several pathophysiological processes; among others, sepsis [3, 5, 6] and the use of controlled MV [7–9], have been identified as triggers of this phenomenon, emphasizing distinct but coexistent local and systemic pathological features. Along with diaphragm dysfunction, peripheral skeletal muscle wasting occurs very early in critically ill patients [10], resulting from a suggested imbalance between a lower muscle protein synthesis and an overwhelming degradation [10, 11]; low skeletal muscle area (assessed by CT scan at L3 level) appears to be an independent risk factor for mortality in mechanically ventilated critically ill patients, independent of sex or ICU admission severity score [12]. Similar mechanisms for diaphragm and peripheral muscle dysfunction in critically ill patients have already been described in the literature. In this context, sepsis and septic shock seem to have an important role by inducing a myopathy through reductions in force-generating capacity, atrophy, and altered bioenergetics [13].

Ultrasonography (US) is a bedside, radiation-free technology, widely used in the ICU setting. In the last two decades, some solutions have emerged in regard to its application for the assessment of the diaphragm (Diaphragm US, DUS) [2, 14–20] and the peripheral muscles (Peripheral Muscle US, PMUS) [21–27]. Shear-Wave Elastography (SWE) applied to the skeletal muscle is an even more recent and promising US technique. Recently, this application has been comprehensively reviewed elsewhere [28]. Already used in other domains, it provides information on muscle “stiffness” (or “hardness”) [28]. SWE application to the diaphragm assessment has already been tested in healthy and critically ill patients [29–31], but its applicability and clinical significance in the follow-up of diaphragm dysfunction in ICU patients need further investigation. Similar, to the aforementioned application, SWE, which is a reliable technique, may be of great interest for the understanding of peripheral muscle impairment and disease [32]. It has been shown to be capable of detecting peripheral muscle changes in Duchenne muscular dystrophy and cerebral palsy patients [33–36]. Ultrasonography might also be useful in mapping the inter-relationship between both diaphragm and peripheral muscle weakness, and their impact on clinical outcomes such as weaning success/failure. In this regard, data about the possible coexistence of these two features and their inter-dependency remains very limited and to our knowledge, no study using a combined approach of DUS and PMUS in the prediction of weaning success/failure has ever been performed. Finally, the relationship between muscle US markers and the muscle contractility remains a topic of interest in the field.

The current clinical protocol “Diaphragm dysfunction and peripheral muscle wasting in septic shock patients: exploring their relationship over time using ultrasound technology (*MUSiShock*)” (ClinicalTrials.gov identifier NCT04550143) is intended to investigate the clinical course of both respiratory and limb muscle atrophy and dysfunction in critically ill patients admitted to the ICU with septic shock. With both physiological and clinical perspectives, it aims to help in the understanding and mapping of processes leading to an early and rapid skeletal muscle wasting and dysfunction in ICU patients as they are rationally associated with poor clinical outcomes, increased morbidity/mortality, and a poor quality of life. An early focused muscle function monitoring may therefore help clinicians to better understand different profiles of muscle weakness during and/or as a consequence of an ICU stay, thereby helping clinicians to implement of measures to counteract it.

In the *MUSiShock* clinical study, we will follow the changes occurring in the diaphragm and quadriceps *rectus femoris* muscles. We aim to answer the following questions:

1. Is there an association between Classic Muscle Ultrasound (CMUS) markers and SWE assessment, when applied to the diaphragm or quadriceps *rectus femoris* muscles?
2. When assessing concurrently the diaphragm and the peripheral muscles, is it possible to find similar behaviors of muscle wasting for both muscle types in any ultrasound marker (SWE included)?
3. Whatever the US method used (SWE or CMUS), can a combined DUS and PMUS approach be more powerful in the prediction of weaning outcomes than DUS alone?

Here, CMUS is used to describe some well-established US markers that can be easily found in the intensive care related scientific literature (thickness and thickness fraction for the diaphragm, echogenicity and cross-sectional area for the peripheral muscles).

## Methods

The following study protocol was prepared according to the STrengthening the Reporting of OBservational studies in Epidemiology (STROBE) recommendations [37, 38]. A STROBE checklist can be found as additional file.

### Study design

*MUSiShock* is single-centre, investigator-initiated (supported by the Geneva University Hospitals, Switzerland), observational prospective cohort study, intended to investigate diaphragm and peripheral muscles through the use of ultrasound techniques in septic shock patients. Participants will be assessed during their ICU stay. The anticipate sample size is of eighty-four participants. Inclusion of patients started in October/2020 and is intended to run for 24 months.

### Approvals

This research project was approved by the local ethical committee (ID 2020–00452, 28 July 2020), the Geneva Ethics Committee (*Commission Cantonale d’Ethique de la Recherche*, CCER); ethical standards of the Declaration of Helsinki will be respected, informed consent will be obtained from participants or their legal representative and confidentiality will be maintained during the research protocol. A study registration was made at clinicaltrials.gov (identifier NCT04550143).

### Setting

Recruitment will be performed at the Geneva University Hospitals (HUG, Switzerland), in a mixed ICU (admitting medical, neurological, trauma, cardiac and surgical patients) with 32 beds, admitting 100 to 130 patients diagnosed with septic shock *per* year. All patients admitted to the study ICU will be screened within the first 24 hours after ICU admission; screening will be performed by investigators and the department’s research nursing team during working days.

All patients admitted to the study ICU will be screened within the first 24 hours after ICU admission; screening will be performed by investigators and the department’s research nursing team.

If eligibility conditions are met, participants (if awaken and able to collaborate) will be approached to provide informed consent within the first 24 hours. Then, data will start to be collected.

For participants not able to provide consent (the expected majority), with permission granted from the medical team in charge (a physician who is not participating in the research project), data will be collected according to the time-points of study procedures. When full collaborative state is regained, each participant’s consent (retrospectively) will be obtained as soon as possible.

In case of death or long-term lack of capacity as consequence of the current critical illness, the next of kin/legal representative will be asked to provide informed consent as soon as possible. In the case where full collaborative state is regained and the participant does not provide informed consent, his/her participation in the project will be immediately closed and all health-related personal data collected up to this moment will be destroyed. The same measures will be applied to the cases where the next to kin/legal representative provided consent (where initially, the full recovery was not anticipated by the medical team), but yet the participant regains full collaborative state and then refuses to consent. For those situations, where the participant or the next to kin/legal representative wishes to withdraw from the project after given a first post hoc consent, data collected up to the withdrawal will be anonymised without delay and kept for analysis to not compromise the study, as stated in the signed informed consent form.

### Timeline

**Fig 1:**
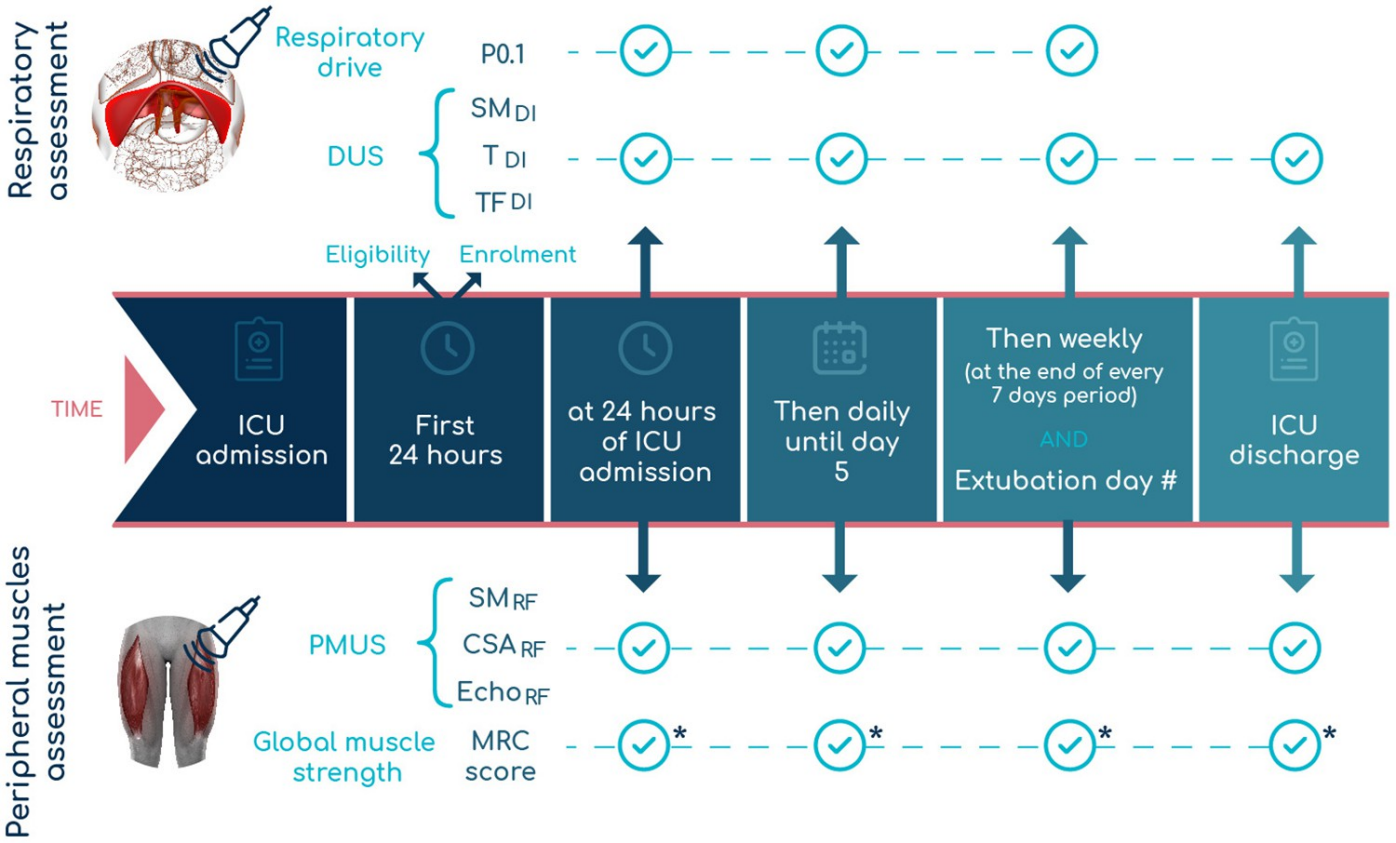
Time-points of assessment for the protocol. *if “awakening” criteria are met. #If taking place after the first 5 days. Abbreviations: CSA_RF_−rectus femoris cross-sectional area; DUS–diaphragm ultrasound; Echo_RF_−rectus femoris echogenicity; ICU–intensive care unit; MRC–medical research council; P0.1 –airway occlusion pressure; PMUS–peripheral muscle ultrasound; SM_DI_−diaphragm shear modulus; SM_RF_−rectus femoris shear modulus; T_DI_−diaphragm thickness; TF_DI_−diaphragm thickness fraction.

The included participants will be assessed at the following time-points (Fig 1):

- at 24 hours of ICU admission
- daily until day 5
- weekly after day 5
- at the extubation day
- at ICU discharge.

### Participants

The inclusion and exclusion criteria for being admitted to the study are presented below.

The inclusion criteria are:

- adult patients (> 18 years old) admitted to the ICU,
- with a diagnosis of septic shock (as defined by the Third International Consensus Definitions, Task Force, Sepsis-3 [39], and integrated in the Surviving Sepsis Campaign guidelines [40]),
- a SOFA score (Sequential Organ Failure Assessment score) equal to or greater than 8 points at ICU admission,
- blood lactate concentration above 2 mmol/L at ICU admission,
- expected to have more than 48h of mechanical ventilation,
- expected to have more than 5 days of ICU length of stay (estimated by the attending physician),
- able to walk prior to ICU admission (walking aids accepted).

The exclusion criteria are:

- pregnancy,
- lower limb amputation, external fixators or open wounds,
- thoracic external fixators or open wounds,
- diagnosed neuromuscular or central nervous system diseases,
- being transferred from another ICU,
- spinal cord injury,
- diaphragm pacemaker,
- palliative goals of care,
- sarcopenia (defined as “a syndrome of its own characterized by the progressive and generalised loss of skeletal muscle mass, strength and function (performance) with a consequent risk of adverse outcomes” [41]),
- cachexia (defined as ““a complex metabolic syndrome associated with underlying illness and characterized by loss of muscle mass with or without loss of fat mass. The prominent feature of cachexia is weight loss in adults. The cachectic phenotype is characterized by weight loss, reduced Body Mass Index (BMI) and reduced muscle mass and function in combination with an underlying disease that displays biochemical indices of on-going elevated inflammatory activity” [41]),
- anorexic disorders (protein-energy malnutrition),
- intellectual or cognitive impairments, limiting the ability to follow instructions.

### Outcome measures

Primary outcome,

**1)** To characterize the association between SWE and other US markers for each muscle (diaphragm and quadriceps *rectus femoris* muscle).

As secondary outcomes,

**2.a)** The association between the changes in DUS and PMUS over time (between different times points and first assessment at ICU admission) will also be explored using change in all US markers.
**2.b)** The analysis of a combined model of one DUS marker and one PMUS marker (multiple combinations will be performed between all markers) to predict weaning success/failure will be performed. In the hosting ICU, eligibility to begin ventilator weaning process is sought systematically and at least twice a day. The criteria for starting the weaning process will follow institutional procedures and rationale, in agreement with international published standards [1].
  In this regard, weaning failure is defined as follows: “the failure of spontaneous breathing trial (SBT) or the need for reintubation within 48 hours following extubation” [1]. Decision to reintubate is made by the attending physician, under an institutional procedure as well.

### Data source/measurements

At each of these time points, we will assess quadriceps *rectus femoris* (RF) and diaphragm muscles (bilaterally), through PMUS and DUS measurements, respectively; while intubated and ventilated, the patient’s airway occlusion pressure (P_0.1_) will be monitored and recorded using the patient’s ventilator. In addition, for all time-points, patients’s ability to collaborate will be “checked” with “awakening” criteria by using the Standardized Five Questions (S5Q) [42]. If this ability to collaborate is confirmed, a Medical Research Council (MRC) sum score assessment will also be performed, which is a recognized and frequently used test in the ICU. Screening and assessments for each time-point will be performed without interfering with sedation/neuromuscular blocking agent medical administration.

All collected data will be prepared according to published standard protocols in the field and will be performed by an experienced team in muscle ultrasound (a physiotherapist and an intensivist), both currently working in the ICU.

US assessments will be performed with as minimal compression as possible and with a copious amount of gel, in order to avoid any deformation in the assessed structure. Probe positions will be marked on the skin in order to guarantee the good repeatability of measurements between each time-point. Offline US measurements (i.e., subsequent calculations performed on the recordings obtained at the bedside) will be performed by the same investigator. Assessments and their set ups are described in detail here bellow.

#### Diaphragm ultrasound (DUS)

We will use a B-mode/M-Mode ultrasound (SuperSonic Imagine, Aix-en-Provence, France). Participants will be in semi-recumbent position (head elevation between 30° and 45°) with knees extended in neutral position and will be asked to maintain his/her own breathing pattern if awaked.

Assessments of diaphragmatic *thickness* (T_DI_), *thickness fraction* (TF_DI_) and SWE derived *shear modulus* (SM_DI_), will be performed for both right and left hemi-diaphragms.

One landmark and one type of probe will be used to obtain these assessments. We will follow the instructions already described in the literature [14, 15]: using a 10- to 2-MHz linear transducer array (SL10-2; SuperSonic Imagine) between the 8^th^ and 10^th^ intercostal space in the mid-axillary or antero-axillary line, 0.5–2 cm below the costophrenic sinus. Two parallel echogenic layers can be easily identified: the nearest line is the parietal pleura, the deeper one is the peritoneum; the diaphragm is the less echogenic structure between these two lines; T_DI_ (cm) will be the calculated as the distance between the two lines at the end of expiration and TF_DI_ as the rate of change between end-expiration and end-inspiration thicknesses (TF_DI_ = “thickness at end-inspiration”–“thickness at end-expiration” / “thickness at end-expiration”, %). Both T_DI_ and TF_DI_ present good reproducibility; an Intraclass Correlation Coefficient (ICC) ranging from 0.876 to 0.999 (intra-observer) and from 0.97 to 0.989 (inter-observer) for T_DI_, and an ICC ranging from 0.876 to 0.98 (intra-observer) and from 0.56 to 0.989 (inter-observer) for TF_DI_ [14].

Shear-wave elastography (SWE) technique is based on Hooke’s law which proposes a relationship between strain, stress and elasticity (s = E∙d, where *s* is the stress, *E* the Young’s modulus, and *d* the deformation or strain). SWE technique assess directly the shear wave velocity which is then used to assess the principal marker defined as Shear Modulus (SM), providing information on muscle “stiffness” (or “hardness”) [28], with results of the last being retrieved in kilopascals (kPa). For SM_DI_ assessment, the ultrasound machine will be turned to the “Shear-wave mode” and an identical probe position will be used. For each image, a region of interest (ROI) covering the widest possible surface of diaphragm (i.e., between the two parallel echogenic layers) and allowing an acquisition frequency of 2Hz will be set (or near this value if technically not possible to achieve it due to the diaphragm depth). Then, inside the pre-recorded ROI, in the offline analysis we will manually trace the contour of the “structure of interest” (Q-box Trace); results will be retrieved in kilopascals (kPa). Reproducibility of the SM_DI_ measurement in critically ill patients was recently published, showing excellent intra- and inter-observer ICCs above 0.92, for the diaphragm application [32].

At the bedside, several real-time clips of 10-s will be acquired. Breath-by-breath analysis will be performed and three images for each T_DI_, TF_DI_, and SM_DI_, will be recorded. The mean value for each hemi-diaphragm and for both will be taken to analysis.

#### Peripheral muscle ultrasound (PMUS)

For the PMUS assessment, the same equipment, probe and participant position used in the DUS, will be replicated. Participants will be asked to stay as relaxed as possible if awake.

To represent peripheral skeletal muscle, the chosen structure to assess will be the quadriceps *rectus femoris* muscle mostly due to his easy accessibility; its limits were defined as the area between the anterior superficial aponeurosis and the central aponeurosis that borders with quadriceps *vastus intermedius* muscle. Assessments of quadriceps *rectus femoris* muscle *cross-sectional area* (CSA_RF_), *echogenicity* (Echo_RF_) and SWE derived *shear modulus* (SM_RF_) will be performed.

We will use the landmarks previously described by Gruther and co-workers [26] and later tested by Tillquist and colleagues in healthy subjects, showing excellent intra- and inter-observer reliability in the assessment of muscle layer thickness (intra- and inter-observer ICCs of 0.98 and 0.95, respectively) [43]. The probe will be placed perpendicularly to the anterior plane of the thigh, in two anatomical points, determined, as follows:

1. in the midpoint between the anterior superior iliac spine and the upper pole of the patella and,
2. the border of the lower third and upper two-thirds between the anterior superior iliac spine and the upper pole of the patella.

CSA_RF_ will be calculated by outlining the area under the muscle’s hyperechoic line (aponeurosis), using the equipment’s cursor on a frozen image. US settings’ adjustments will always be performed in order to improve quality of the image acquisition. Values will be retrieved in cm^2^.

For Echo_RF_ (differences in grey-scale images), settings of the first assessment will be recorded and kept for the following time-points evaluation. A further quantitative grey-scale analysis (ranging between 0 and 255, black = 0 and white = 255) in a pre-determined ROI of 2cm x 2cm will be performed, using ImageJ software (National Institutes of Health, USA) [44]. If not possible, we will use the largest square size within the anatomical boundaries of the muscle. Results will be retrieved in mean grey-scale (ranging from 0 to 255). Good reproducibility was shown previously for both CSA_RF_ (inter-observer correlation coefficient of 0.97) and Echo_RF_ (inter-image correlation coefficient of 0.94) measurements [10, 45].

Similar to the technical approach described to the diaphragm, SWE derived SM_RF_ will assess *rectus femoris* muscle’s stiffness. Excellent reproducibility was shown as well for its *rectus femoris* application (intra- and inter-observer ICCs above 0.91) [32].

*Per* landmark, three images for CSA_RF_, Echo_RF_, and SM_RF_, will be recorded. The mean for each landmark and for each limb (the average for the two landmarks) will be taken to analysis.

#### Airway occlusion pressure (P_0.1_)

P_0.1_ is “the pressure developed in the occluded airway 100 milliseconds after the onset of inspiration” [46]. Its use doesn’t require any additional equipment since it can be easy measured by using patient’s ventilator.

For the measurement itself, the same position and the same commands as used in the DUS and PMUS assessment will be applied. After 5 minutes breathing without any interruption or disturbance, 4 measurements will be observed and recorded as displayed on the ventilator screen.

The average of all recorded repetitions will be brought to analysis. Results will be expressed in cmH_2_O.

#### Standardized Five Questions (S5Q)

The criterion is based on 5 commands, as follows: “open/close your eyes”, “look at me”, “open your mouth and stick out your tongue”, “nod your head” and “raise your eyebrows when I have counted to 5” [42, 47]. The minimal score of 3 on 5 questions will identify the patient as “able to collaborate”.

#### Medical Research Council (MRC) sum score

This is a manual muscle strength testing tool, used very often in the ICU setting. It’s based on the assessment of the following muscle groups: shoulder abduction, elbow flexion, wrist extension, hip flexion, knee extension, and dorsiflexion of the ankle, all scored bilaterally [48–50]. Muscle strength is graded as follows: **0**, “no visible/palpable contraction”; **1**, “visible/palpable contraction without movement of the limb”; **2**, “movement of the limb, but not against gravity”; **3**, “movement against gravity”; **4**, “movement against gravity and some resistance”; **5**, “normal” [49, 51]. High values of ICCs, above 0.94, have already been reported in the literature in regard to interobserver agreement for the MRC sum score [52, 53].

The sum score ranges between 0 and 60 (between 0 and 5, in 12 muscle groups), with a score <48 indicating the presence of weakness [47]. The sum score values will be taken for analysis.

Other clinical data will be recorded, as far as possible, exploiting existing systems, such as critical care information system and hospital’s electronic medical records database including the annotation of descriptive data (demographics medical history; concomitant medication; laboratory analysis; medical events; hospitalisation and interventions and adverse events). Other information from the ICU’ current practice such as the respiratory system status, cardiovascular and hemodynamics, nutrition, drugs and therapies administered, biological tests, length of stay (LOS) and mortality, among others, will be retrieved through the use of this system as well.

After completing all the assessment for each time-point, coded data will then be uploaded to an electronic Case Report Form (eCRF) into a dedicated Clinical Database Management System (CDMS) software program.

### Sample size estimation

For these analyses, we aim to be able to identify a linear correlation between the two US markers (SWE and one CMUS) and between the 2 muscles examined for each time-point. For that, we estimated a medium effect size of 0.30 for Pearson’s r, as proposed by Cohen [54]. To reach the power to identify such an association with 80% power, an alpha level of 5% and a 2-sided test, we would need to include a minimum of 84 participants.

G*Power v.3.1.9.3 [55] was the software used to perform these calculations.

### Statistical methods

Descriptive statistics will be performed to describe the sample characteristics and to report outcome results. Variables will be presented as mean (and standard deviation) or median (and interquartile range), depending on whether normal distribution assumption is verified or not, respectively.

Changes over time in DUS markers, PMUS markers and MRC sum score will be explored using repeated-measures analysis of variance (ANOVA) or the Friedman test.

For each time-point, association between different DUS or PMUS marker (DUS *versus* DUS or PMUS *versus* PMUS) will be analysed using *Pearson’s* or *Spearman’s Rank-Order* correlations tests; associations between DUS and PMUS (one DUS to one PMUS markers), will be assessed as well. Bland-Altman analysis will be performed to quantify the agreement (diagnostic accuracy) between US techniques for each structure.

In the prediction of weaning success and the presence of ICU-Acquired Weakness (ICUAW), logistic regression models will be performed taking into account the percentage of change (in relation to ICU admission values) and the absolute values of DUS markers alone, PMUS markers alone, and combined DUS and PMUS markers; these models will be adjusted for measured covariates (when available) and will also include reasons for extubation success/failure other than muscle weakness (i.e., those related to airway and non-airway failure).

Furthermore, discriminative power of all ultrasonographic techniques to detect weaning/failure will be assessed using Receiver Operating Characteristics (ROC) curves and Area Under the Curve (AUC) calculations.

All results will be reported at 95% confidence interval. Two-tailed p-values at 0.05 will be considered statistically significant. SPSS v.24.0 (SPSS, Chicago, Illinois, USA) and Stata software v.14.0 (StataCorp LLC, College Station, Texas, USA) will be the software used for statistical analysis.

## Discussion

This research project aims to focus on critically ill patients, who are the sickest among those of a hospital population using radiation-free and user-friendly technology. Critical illness can have a major impact on respiratory muscles, since most patients need mechanical respiratory support, and on locomotor muscles, as patients remain sedated and thus bedridden for several days/weeks. Moreover, in the septic shock patient, this is even more true since this pathophysiological entity generates greater systemic catabolic processes at the muscle level. Their impact on a delay or failure in weaning from respiratory support and physical functionality at ICU discharge are major concerns. An early and accurate assessment of muscle health is, therefore, highly relevant. A better and earlier detection of muscle wasting may also have the potential to help clinicians in the anticipation of clinically relevant questions such as the short-/long-term respiratory and physical function outcomes in septic shock patients. Anticipating these short-term outcomes related to weaning and muscle weakness could also be socially important, since ICU survivors have higher use of health care resources which entails great financial costs.

## Trial status

*MUSiShock* is currently recruiting patients. Human resources reallocation due to the new SARS-COV-2 pandemic forced a project suspension from November/2020 to January/2021. It was resumed in February/2021.

Up to the submission of this protocol, seven participants were included and completed the assessment follow-up process until ICU discharge. This study is expected to be completed in the end of 2022/beginning of 2023.

## Supporting information

S1 File STROBE checklist. (DOC)

S2 File Executive summary. (DOC)

## Abbreviations

ANOVA: Analysis Of Variance
AUC: Area Under the Curve
BMI: Body Mass Index
CDMS: Clinical Database Management System
CMUS: Classic Muscle Ultrasound
CRF: Case Report Form
CSA: Cross-Sectional Area
CSA_RF_: Rectus Femoris Cross-Sectional Area
DUS: Diaphragm Ultrasound
Echo_RF_: Rectus Femoris Echogenicity
EX_DI_: Diaphragm Excursion
HUG: Hôpitaux Universitaires de Genève
ICU: Intensive Care Unit
ICU-AW: Intensive Care Unit-Acquired Weakness
kPa: Kilopascals
LOS: Length Of Stay
MRC: Medical Research Council
MV: Mechanical Ventilation
P0.1: Airway Occlusion Pressure
P_DI_: Transdiaphragmatic Pressure
PMUS: Peripheral Muscle Ultrasound
RF: Rectus Femoris
ROC: Receiver Operating Characteristics
ROI: Region Of Interest
S5Q: Standardized Five Questions
SBT: Spontaneous Breathing Trial
SM: Shear Modulus
SM_DI_: Diaphragm Shear Modulus
SM_RF_: Rectus Femoris Shear Modulus
SOFA: Sequential Organ Failure Assessment score
SPSS: Statistical Package for the Social Sciences
STROBE: STrengthening the Reporting of OBservational studies in Epidemiology
SWE: Shear-Wave Elastography
T_DI_: Diaphragm Thickness
TF_DI_: Diaphragm Thickness Fraction
US: Ultrasonography/Ultrasound
