# Supplementary material for: Diaphragm dysfunction and peripheral muscle wasting in septic shock patients: Exploring their relationship over time using ultrasound technology (the MUSiShock protocol)
Source: PLoS One. 2022 Mar 28;17(3):e0266174. doi: 10.1371/journal.pone.0266174 (PMC8959181; doi:10.1371/journal.pone.0266174)
Supplement: S2 File — (DOC) [file pone.0266174.s002.doc]

**Executive summary**

**Study Title**

**“Diaphragm dysfunction and peripheral muscle wasting in septic shock patients: exploring their relationship over time using ultrasound technology (the MUSiShock protocol)”**

**Short Title: MUS**i**S**hock - “Muscle Ultrasound Study in Shock Patient”

**Authors:** Ivo NETO SILVA, José Alberto DUARTE, Aurélie PERRET, Nicolas DOUSSE, Hannah WOZNIAK, Bernardo BOLLEN PINTO, Raphaël GIRAUD, Karim BENDJELID

**ethics approval and registration:** Swissethics - 2020-00452 / ClinicalTrials.gov - [NCT04550143](https://clinicaltrials.gov/ct2/show/NCT04550143)

**Study category and Rationale:** Research projects **not** classified as clinical trial.

**Category A (Swiss law – Human Research Act, HRA, and Human Research Ordinance, HRO) – “**Measures for sampling biological material or for the collection of personal data entail only minimal risks and discomfort”

🡪 This is a prospective observational study intended to investigate muscle wasting in septic shock patients.

**Background and Rationale**

Intensive Care Unit (ICU) patients are known to lose muscle mass and function for many reasons, including prolonged immobilization, the effects of ICU treatments such as mechanical ventilation (MV) and the critical illness itself. Ultrasonography (US) is a bedside, radiation-free technology, widely used in the ICU setting. In the last two decades, new techniques have emerged in regard to its application for the assessment of the skeletal muscle (both limb and thoracic). It is a promising tool that may help detecting early changes in muscle structure and function. *MUSiShock* is a research project aiming to investigate structure and function of diaphragm and peripheral muscles using ultrasound techniques (Diaphragm US, DUS; Peripheral Muscle US, PMUS) in septic shock patients, and to assess their association with clinical outcomes such as the weaning from MV.

**Study design and schedule**

*MUSiShock* is single-centre, investigator-initiated, observational prospective cohort study, intended to investigate diaphragm and peripheral muscles (quadriceps *rectus femoris*) through the use of ultrasound techniques in septic shock patients. Participants will be assessed during their ICU stay. Recruitment period started in October/2020 and is intended to run for 24 months.

**Outcomes**

Primary outcome:

**1)** To characterize the association between Shear-Wave Elastography (SWE) and other US markers (thickness and thickness fraction for the diaphragm, echogenicity and cross-sectional area for the peripheral muscles).

As secondary outcomes:

**2.a)** The association between the changes in DUS and PMUS over time (between different times points and first assessment at ICU admission) will also be explored using change in all US markers.

**2.b)** The analysis of a combined model of one DUS marker and one PMUS marker (multiple combinations will be performed between all markers) to predict weaning success/failure will be performed as well.

**Eligibility**

| **Inclusion criteria** |  | **Exclusion criteria** |
| --- | --- | --- |
| - Adult patients (> 18 years old) admitted to the ICU, - With a diagnosis of septic shock, - A SOFA score equal to or greater than 8 points, at ICU admission, - Blood lactate concentration above 2 mmol/L at ICU admission, - Expected to have more than 48h of mechanical ventilation, - Expected to stay more than 5 days in the unit, by the attending physician, - Able to walk prior to ICU admission (walking aids accepted). |  | - Any underlying chronic disease leading skeletal muscle weakness/paralysis, - Pregnancy, - Lower limb amputation, fixators or open wounds, - Thoracic fixators or open wounds, - Being transferred from another ICU, - Sarcopenia or cachexia, - Palliative goals of care, - Intellectual or cognitive impairments, limiting the ability to follow instructions. |

**Recruitment Procedure**

All patients admitted to the study ICU will be screened within the first 24 hours after ICU admission; screening will be performed by investigators and the department’s research nursing team.

If eligibility conditions are met, participants (if awaken and able to collaborate) will be approached to provide informed consent within the first 24 hours. Then, data will start to be collected.

For participants not able to provide consent (the expected majority), with permission granted from the medical team in charge (a physician who is not participating in the research project, HRA, art. 30), data will be collected according to the study procedures. Participant’s consent (retrospectively) will be obtained as soon as patients regain capacity to provide consent.

In case of death or long-term disability impairing capacity to provide consent, the next of kin/legal representative will be asked to provide informed consent as soon as possible (HRO, art. 11).

**Measurements and procedures**

Study visits will be performed as follows:

- at **24 hours of ICU admission**
- **daily until day 5**, then
- **weekly** (at the end of every 7 days period),
- **at the extubation time**,
- at **ICU discharge**.

At each of these time points, we will assess quadriceps *rectus femoris* (RF) and diaphragm muscles (bilaterally), through PMUS and DUS measurements, respectively; while intubated and ventilated, the patient’s airway occlusion pressure (P_0.1_) will be monitored and recorded using the patient’s ventilator. In addition, for all time-points, patients’s ability to collaborate will be “checked” with “awakening” criteria by using the Standardized Five Questions (S5Q). If this ability to collaborate is confirmed, a Medical Research Council (MRC) sum score assessment will also be performed. Screening and assessments for each time-point will be performed without interfering with sedation/neuromuscular blocking agent medical administration.

**Number of Participants AND Rationale:** 84 participants.

For rationale, please see section “Statistical Considerations”, here bellow.

**Statistical Considerations:**

Changes over time in DUS markers, PMUS markers and MRC sum score will be explored using repeated-measures analysis of variance (ANOVA) or the Friedman test. For each time-point, association between different DUS or PMUS marker (DUS *versus* DUS or PMUS *versus* PMUS) will be analysed using *Pearson’s* or *Spearman’s Rank-Order* correlations tests; associations between DUS and PMUS (one DUS to one PMUS markers), will be assessed as well. Bland-Altman analysis will be performed to quantify the agreement (diagnostic accuracy) between US techniques for each structure.

In the prediction of weaning success and the presence of ICU-Acquired Weakness (ICUAW), logistic regression models will be performed taking into account the percentage of change (in relation to ICU admission values) and the absolute values of DUS markers alone, PMUS markers alone, and combined DUS and PMUS markers; these models will be adjusted for measured covariates (when available). Furthermore, discriminative power of all ultrasonographic techniques to detect weaning/failure will be assessed using Receiver Operating Characteristics (ROC) curves and Area Under the Curve (AUC) calculations.

Sample size estimation

For these analyses, we aim to be able to identify a linear correlation between the two US markers (SWE and one CMUS) and between the 2 muscles examined for each time-point. For that, we estimated a medium effect size of 0.30 for Pearson’s r, as proposed by Cohen. To reach the power to identify such an association with 80% power, an alpha level of 5% and a 2-sided test, we would need to include a minimum of **84 participants**.
